# Supplementary material for: Genome-Wide Identification, Characterization, and Comparative Analysis of NLR Resistance Genes in Coffea spp
Source: Front Plant Sci. 2022 Jul 7;13:868581. doi: 10.3389/fpls.2022.868581 (PMC9301388; doi:10.3389/fpls.2022.868581)
Supplement: Supplementary file 1 [file Data_Sheet_1.zip › Supplementary Figure 1.pdf]

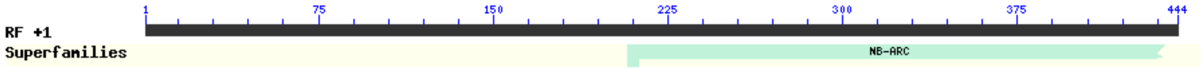

ID	Name	Accession	Description	Interval	E-value
**LOC113737176_Ca**	NB-ARC super family	cl26397	NB-ARC domain	208-438	8.92e-11

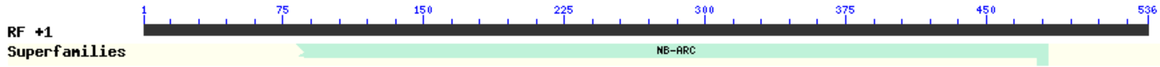

ID	Name	Accession	Description	Interval	E-value
**LOC113735982_Ca**	NB-ARC super family	cl26397	NB-ARC domain	82-483	1.90e-11

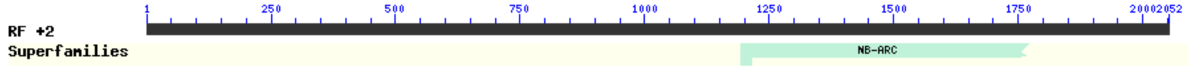

ID	Name	Accession	Description	Interval	E-value
**Cc02_g12220_Cc**	NB-ARC super family	cl26397	NB-ARC domain	1193-1771	3.08e-08

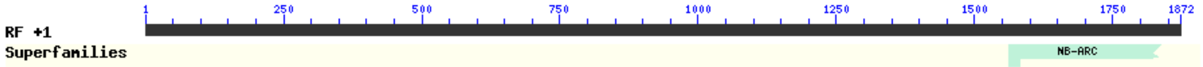

ID	Name	Accession	Description	Interval	E-value
**Cc03_g10360_Cc**	NB-ARC super family	cl26397	NB-ARC domain	1561-1836	7.70e-13

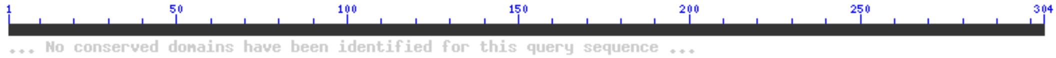

ID	Name	Accession	Description	Interval	E-value
**Cc07_g18800_Cc**	-	-	-	-	-

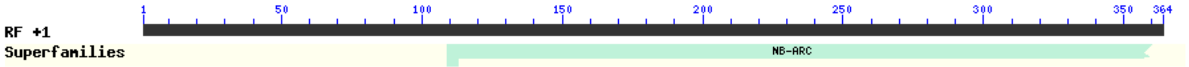

ID	Name	Accession	Description	Interval	E-value
**Cc00_g21910_Cc**	NB-ARC super family	cl26397	NB-ARC domain	109-360	7.62e-08

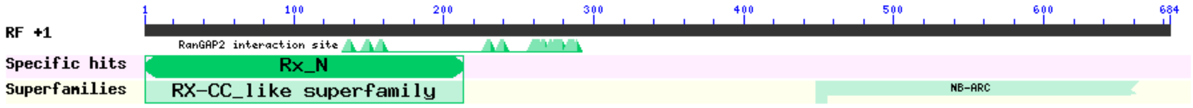

ID	Name	Accession	Description	Interval	E-value
**Cc00_g35420_Cc**	Rx_N	pfam18052	Rx N-terminal domain	1-213	1.29e-17
	NB-ARC super family	cl26397	NB-ARC domain	448-663	1.78e-10

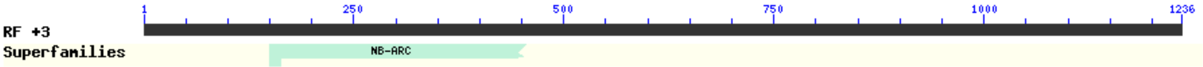

ID	Name	Accession	Description	Interval	E-value
**LOC113766615_Ce**	NB-ARC super family	cl26397	NB-ARC domain	150-455	1.53e-11

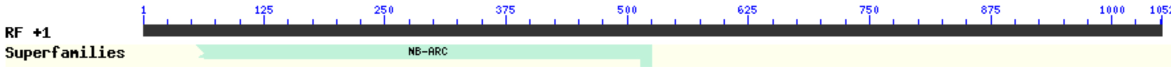

ID	Name	Accession	Description	Interval	E-value
**LOC113766771_Ce**	NB-ARC super family	cl26397	NB-ARC domain	55-525	9.82e-07

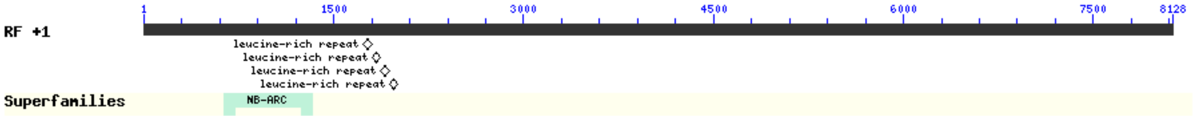

ID	Name	Accession	Description	Interval	E-value
**LOC113766774_Ce**	NB-ARC super family	cl26397	NB-ARC domain	637-1326	9.69e-11

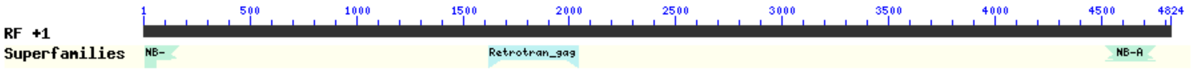

ID	Name	Accession	Description	Interval	E-value
**LOC113777141_Ce**	Retrotran_gag_2 super family	cl26047	gag-polypeptide of LTR copia-type; This family is found in Plants and fungi	1624-2040	2.22e-14
	NB-ARC super family	cl26397	NB-ARC domain	7-165	1.58e-10
	NB-ARC super family	cl26397	NB-ARC domain	4519-4749	7.73e-10

Supplementary Figure 1. **Conserved domains analysis for genes not found by the NLR-annotator in the coffee genomes**. For the analysis, nucleotide sequences were used. The table shows the list of domain hits found for each of the sequences. The conserved domains were detected using NCBI Conserved Domain Database and the graphical summary was set to concise view. If the alignment omitted more than 20% of the either the N- or C-terminus or both, the partial nature of the hit is indicated in the graphical display as domain with jagged edges. At the end of each ID, the source genome is identified, Ca: *C. arabica*, Cc: *C. eugenioides* and Ce: *C. eugenioides*. RF: reading frame.
