# Supplementary material for: Genome-Wide Identification, Characterization, and Comparative Analysis of NLR Resistance Genes in Coffea spp
Source: Front Plant Sci. 2022 Jul 7;13:868581. doi: 10.3389/fpls.2022.868581 (PMC9301388; doi:10.3389/fpls.2022.868581)
Supplement: Supplementary file 1 [file Data_Sheet_1.zip › Supplementary Figure 2.pdf]

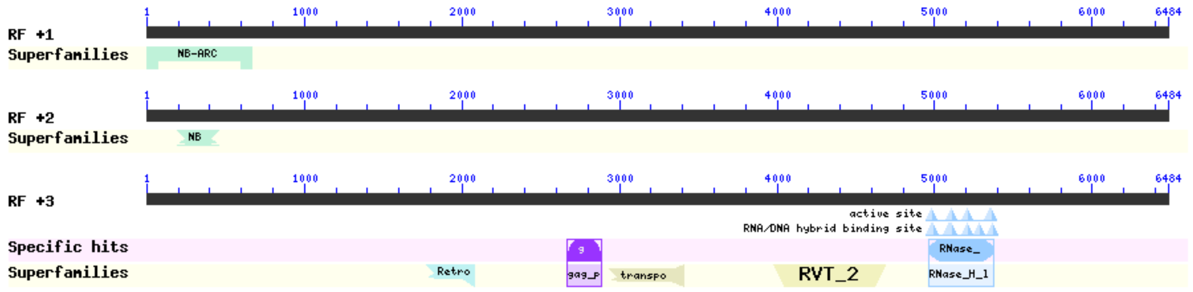

Chr_4c_nlr_22_Ca partial				
**Name**	**Accession**	**Description**	**Interval**	**E-value**
NB-ARC super family	cl26397	NB-ARC domain	1-663	1.67e-06
NB-ARC super family	cl26397	NB-ARC domain	194-457	4.21e-12
RNase_HI_RT_Ty1	cd09272	Ty1/Copia family of RNase HI in long-term repeat retroelements	4965-5378	2.30e-60
RVT_2 super family	cl06662	Reverse transcriptase (RNA-dependent DNA polymerase)	3978-4682	8.87e-51
Retrotran_gag_2 super family	cl26047	gag-polypeptide of LTR copia-type; This family is found in Plants and fungi	1779-2075	5.08e-16
transpos_IS481 super family	cl41329	IS481 family transposase	2934-3404	3.45e-12
gag_pre-integrs	pfam13976	GAG-pre-integrase domain; This domain is found associated with retroviral insertion elements	2670-2885	3.14e-11

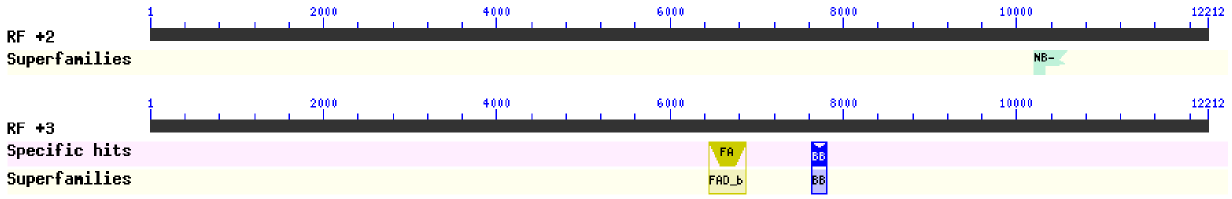

Chr_3c_nlr_44_Ca complete (pseudogene)				
**Name**	**Accession**	**Description**	**Interval**	**E-value**
NB-ARC super family	cl26397	NB-ARC domain	10199-10585	2.48e-10
FAD_binding_4	pfam01565	FAD binding domain; This family consists of various enzymes that use FAD as a co-factor	6453-6872	5.37e-19
BBE	pfam08031	Berberine and berberine like; This domain is found in the berberine bridge and berberine	7632-7805	5.77e-14

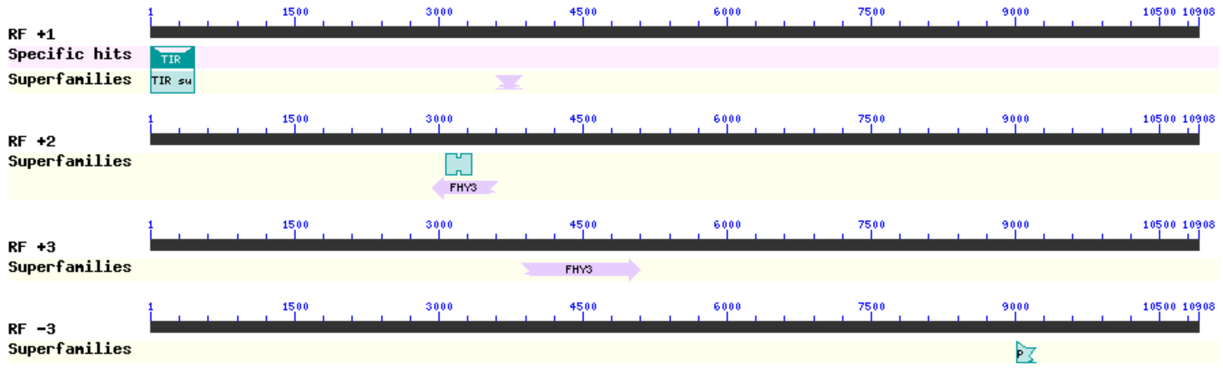

Chr_10c_nlr_18_Ca partial (pseudogene)				
**Name**	**Accession**	**Description**	**Interval**	**E-value**
TIR	pfam01582	TIR domain; The Toll/interleukin-1 receptor (TIR)	4-450	1.05e-54
FHY3 super family	cl31971	Protein FAR-RED ELONGATED HYPOCOTYL 3	3598-3861	5.24e-22
FAR1 super family	cl40636	FAR1 DNA-binding domain	3074-3334	1.24e-19
FHY3 super family	cl31971	Protein FAR-RED ELONGATED HYPOCOTYL 3	2936-3610	7.11e-17
FHY3 super family	cl31971	Protein FAR-RED ELONGATED HYPOCOTYL 3	3861-5096	1.62e-62
PH-like super family	cl17171	Pleckstrin homology-like domain; The PH-like family includes the PH domain	9019-9207	4.83e-08

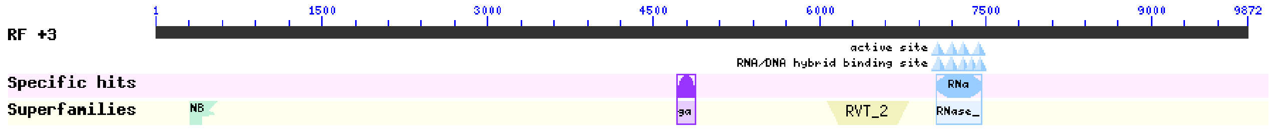

Chr_8c_nlr_13_Ca complete (pseudogene)				
**Name**	**Accession**	**Description**	**Interval**	**E-value**
RVT_2 super family	cl06662	Reverse transcriptase (RNA-dependent DNA polymerase)	6069-6800	2.16e-76
RNase_HI_RT_Ty1	cd09272	Ty1/Copia family of RNase HI in long-term repeat retroelements; Ribonuclease H (RNase H)	7053-7469	3.08e-74
gag_pre-integrs	pfam13976	GAG-pre-integrase domain; This domain is found associated with retroviral insertion elements	4710-4874	1.19e-08
NB-ARC super family	cl26397	NB-ARC domain	309-554	2.54e-06

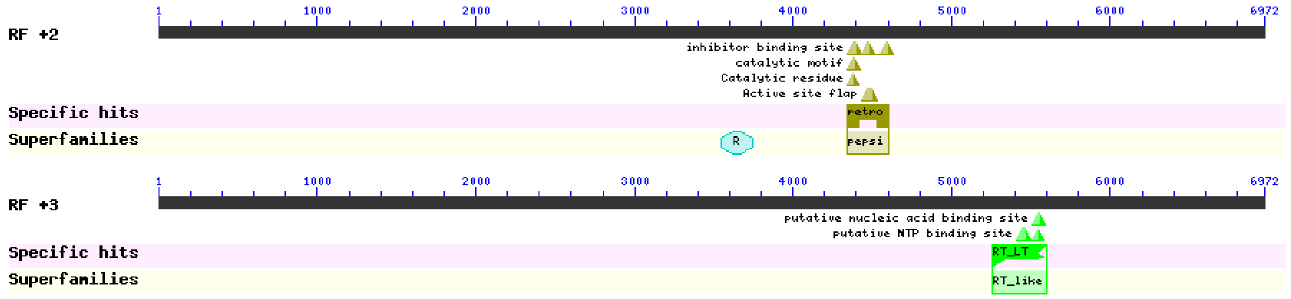

Chr_11c_nlr_73_Ca partial (pseudogene)				
Name	Accession	Description	Interval	E-value
retropepsin_like	cd00303	Retropepsins; pepsin-like aspartate proteases	4346-4606	5.67e-11
Retrotrans_gag super family	cl29674	Retrotransposon gag protein; Gag or Capsid-like proteins from LTR retrotransposons.	3542-3745	1.56e-07
RT_LTR	cd01647	RT_LTR: Reverse transcriptases (RTs)	5256-5597	2.09e-40

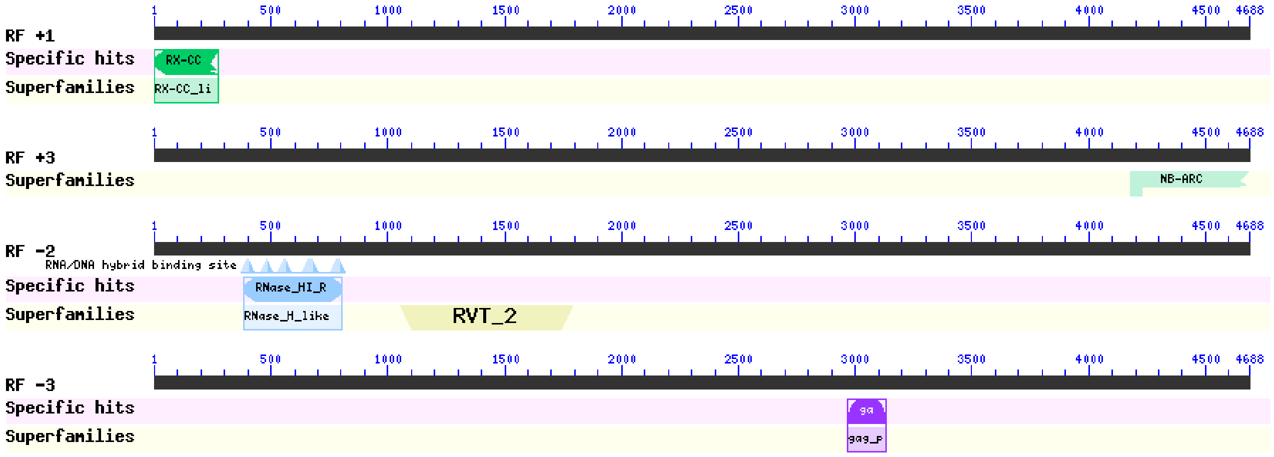

Chr_6e_nlr_10_Ca partial (pseudogene)				
Name	Accession	Description	Interval	E-value
RX-CC_like	cd14798	Coiled-coil domain of the potato virus X resistance protein and similar proteins	1-273	3.80e-15
NB-ARC super family	cl26397	NB-ARC domain	4176-4685	2.49e-12
RNase_HI_RT_Ty1	cd09272	Ty1/Copia family of RNase HI in long-term repeat retroelements; Ribonuclease H (RNase H)	386-802	4.93e-78
RVT_2 super family	cl06662	Reverse transcriptase (RNA-dependent DNA polymerase)	1055-1786	1.73e-68
gag_pre-integrs	pfam13976	GAG-pre-integrase domain; This domain is found associated with retroviral insertion elements	2965-3132	1.08e-06

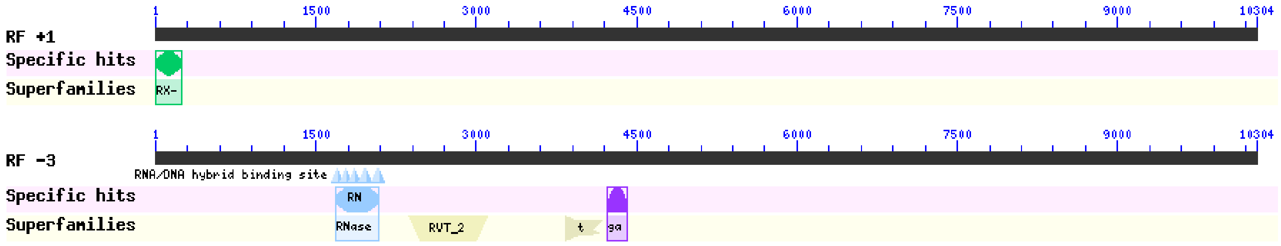

Chr_1e_nlr_61_Ca complete (pseudogene)				
Name	Accession	Description	Interval	E-value
Rx_N	pfam18052	This entry represents the N-terminal domain found in many plant resistance proteins	1-243	9.30e-08
RVT_2 super family	cl06662	Reverse transcriptase (RNA-dependent DNA polymerase)	2365-3102	1.26e-70
RNase_HI_RT_Ty1	cd09272	Ty1/Copia family of RNase HI in long-term repeat retroelements; Ribonuclease H (RNase H)	1678-2091	3.28e-64
transpos_IS481 super family	cl41329	IS481 family transposase	3832-4170	7.73e-17
gag_pre-integrs	pfam13976	GAG-pre-integrase domain; This domain is found associated with retroviral insertion elements	4222-4410	1.42e-12

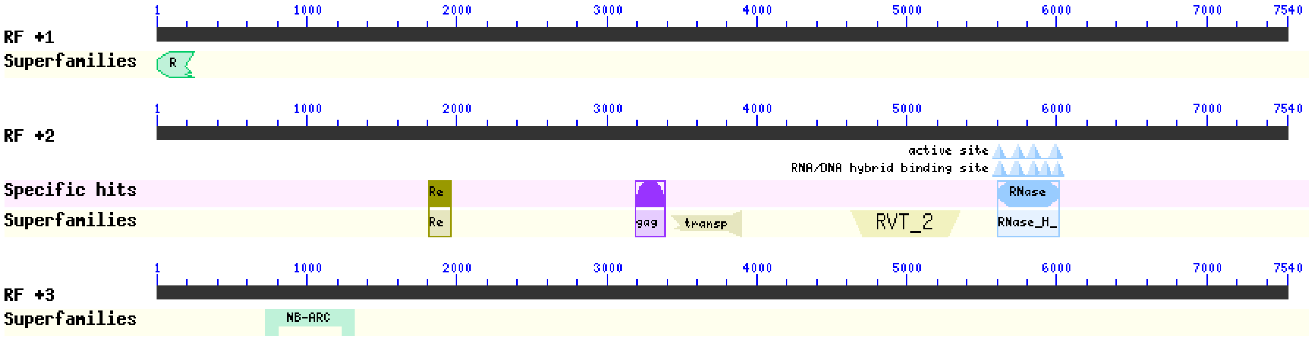

Chr_11e_nlr_43_Ca complete (pseudogene)				
Name	Accession	Description	Interval	E-value
RX-CC_like super family	cl36576	Coiled-coil domain of the potato virus X resistance protein and similar proteins	1-249	9.15e-09

RVT_2 super family	cl06662	Reverse transcriptase (RNA-dependent DNA polymerase)	4622-5350	3.81e-78
RNase_HI_RT_Ty1	cd09272	Ty1/Copia family of RNase HI in long-term repeat retroelements; Ribonuclease H (RNase H)	5600-6019	4.36e-77
Retrotran_gag_3	pfam14244	gag-polypeptide of LTR copia-type; This family is found in Plants and fungi	1814-1954	4.42e-15
transpos_IS481 super family	cl41329	IS481 family transposase	3431-3889	2.46e-10
gag_pre-integrs	pfam13976	GAG-pre-integrase domain; This domain is found associated with retroviral insertion elements	3191-3382	1.51e-08
NB-ARC super family	cl26397	NB-ARC domain	723-1313	1.69e-39

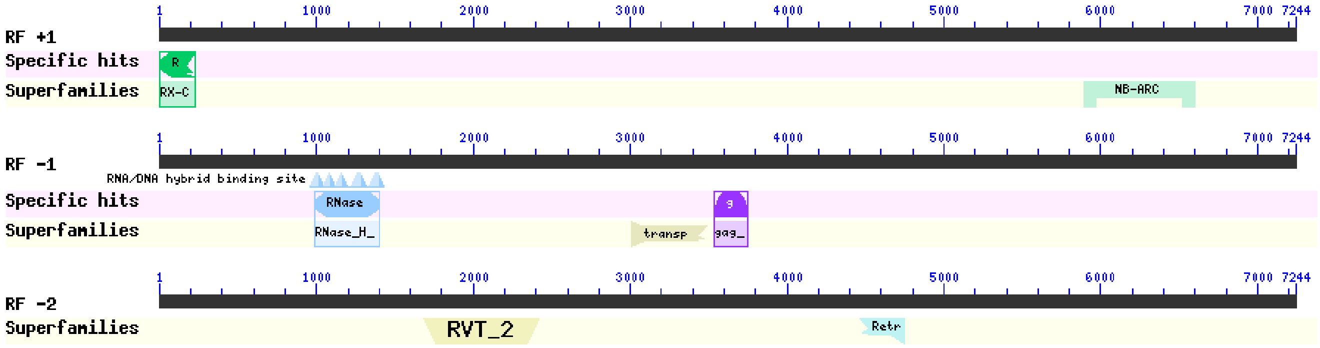

Chr_10e_nlr_2_Ca complete (pseudogene)				
**Name**	**Accession**	**Description**	**Interval**	**E-value**
NB-ARC super family	cl26397	NB-ARC domain	5896-6600	5.29e-44
RX-CC_like	cd14798	Coiled-coil domain of the potato virus X resistance protein and similar proteins	1-225	1.03e-16
RNase_HI_RT_Ty1	cd09272	Ty1/Copia family of RNase HI in long-term repeat retroelements; Ribonuclease H (RNase H)	993-1400	8.74e-66
gag_pre-integrs	pfam13976	GAG-pre-integrase domain; This domain is found associated with retroviral insertion elements	3534-3746	6.29e-12
transpos_IS481 super family	cl41329	IS481 family transposase	3012-3482	7.08e-11
RVT_2 super family	cl06662	Reverse transcriptase (RNA-dependent DNA polymerase)	1679-2419	1.18e-48
Retrotran_gag_2 super family	cl26047	gag-polypeptide of LTR copia-type; This family is found in Plants and fungi	4460-4741	2.61e-09

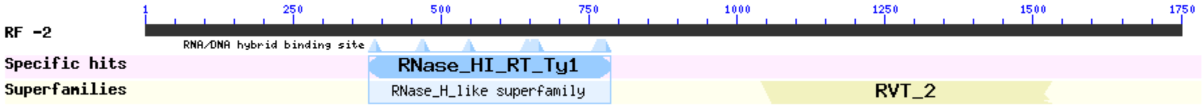

chr0_nlr_300_Cc partial (pseudogene)				
Name	Accession	Description	Interval	E-value
RNase_HI_RT_Ty1	cd09272	Ty1/Copia family of RNase HI in long-term repeat retroelements; Ribonuclease H (RNase H)	378-785	3.94e-67
RVT_2 super family	cl06662	Reverse transcriptase (RNA-dependent DNA polymerase)	1041-1529	4.39e-47

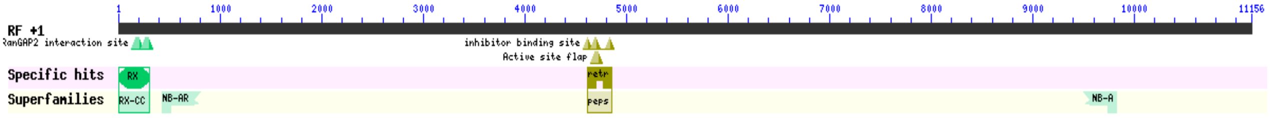

chr8_nlr_26_Cc complete (pseudogene)				
Name	Accession	Description	Interval	E-value
NB-ARC super family	cl26397	NB-ARC domain	427-801	1.88e-22
NB-ARC super family	cl26397	NB-ARC domain	9502-9828	2.21e-15
RX-CC_like	cd14798	Coiled-coil domain of the potato virus X resistance protein and similar proteins	4-300	2.90e-11
retropepsin_like	cd00303	Retropepsins; pepsin-like aspartate proteases; The family includes pepsin-like aspartate proteases from retroviruses, retrotransposons and retroelements	4612-4851	3.61e-06

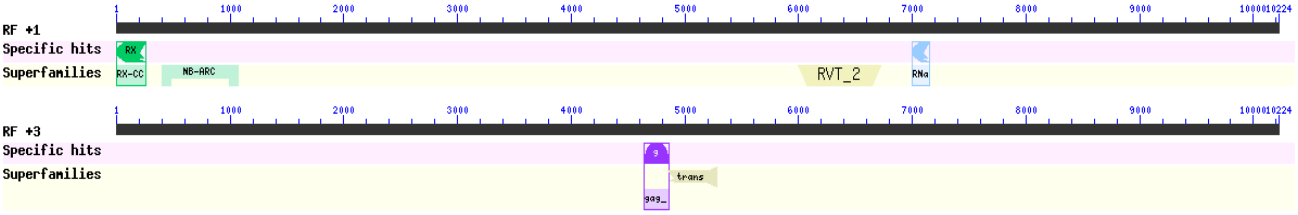

chr7_nlr_33_Cc complete (pseudogene)				
Name	Accession	Description	Interval	E-value
RVT_2 super family	cl06662	Reverse transcriptase (RNA-dependent DNA polymerase)	5992-6726	3.87e-67
NB-ARC super family	cl26397	NB-ARC domain	403-1068	1.85e-50
RNase_HI_RT_Ty1	cd09272	Ty1/Copia family of RNase HI in long-term repeat retroelements; Ribonuclease H (RNase H)	7000-7149	1.94e-19
RX-CC_like	cd14798	Coiled-coil domain of the potato virus X resistance protein and similar proteins	1-261	1.47e-11
transpos_IS481 super family	cl41329	IS481 family transposase	4848-5276	1.29e-14
gag_pre-integrs	pfam13976	GAG-pre-integrase domain; This domain is found associated with retroviral insertion elements	4638-4862	3.10e-12

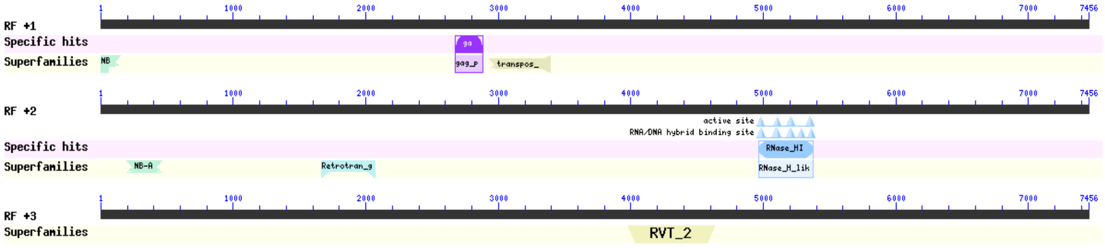

chr4_nlr_22 Cc complete				
Name	Accession	Description	Interval	E-value
transpos_IS481 super family	cl41329	IS481 family transposase	2935-3393	1.65e-13
gag_pre-integrs	pfam13976	GAG-pre-integrase domain; This domain is found associated with retroviral insertion elements	2671-2886	4.35e-11
NB-ARC super family	cl26397	NB-ARC domain	1-147	8.67e-07
RNase_HI_RT_Ty1	cd09272	Ty1/Copia family of RNase HI in long-term repeat retroelements; Ribonuclease H	4964-5377	1.25e-52
Retrotran_gag_2 super family	cl26047	gag-polypeptide of LTR copia-type; This family is found in Plants and fungi	1664-2068	6.38e-22
NB-ARC super family	cl26397	NB-ARC domain	194-457	4.89e-12
RVT_2 super family	cl06662	Reverse transcriptase (RNA-dependent DNA polymerase)	3978-4631	7.19e-46

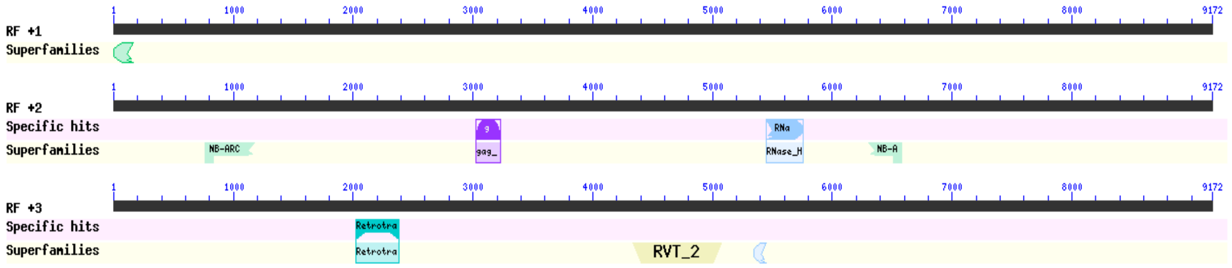

**Chr_5_nlr_92_Ce complete**				
**Name**	**Accession**	**Description**	**Interval**	**E-value**
RX-CC_like super family	cl36576	Coiled-coil domain of the potato virus X resistance protein and similar proteins	1-165	1.40e-07
RNase_HI_RT_Ty1	cd09272	Ty1/Copia family of RNase HI in long-term repeat retroelements; Ribonuclease H	5450-5752	1.79e-44
NB-ARC super family	cl26397	NB-ARC domain	767-1171	1.40e-26
gag_pre-integrs	pfam13976	GAG-pre-integrase domain; This domain is found associated with retroviral insertion elements	3026-3229	8.44e-13
NB-ARC super family	cl26397	NB-ARC domain	6305-6580	5.76e-09
RVT_2 super family	cl06662	Reverse transcriptase (RNA-dependent DNA polymerase)	4332-5072	3.19e-69
Retrotran_gag_2	pfam14223	gag-polypeptide of LTR copia-type; This family is found in Plants and fungi	2022-2381	3.58e-31
RNase_H_like super family	cl14782	Ribonuclease H-like superfamily, including RNase H, HI, HII, HIII, and RNase-like domain IV	5343-5444	2.92e-08

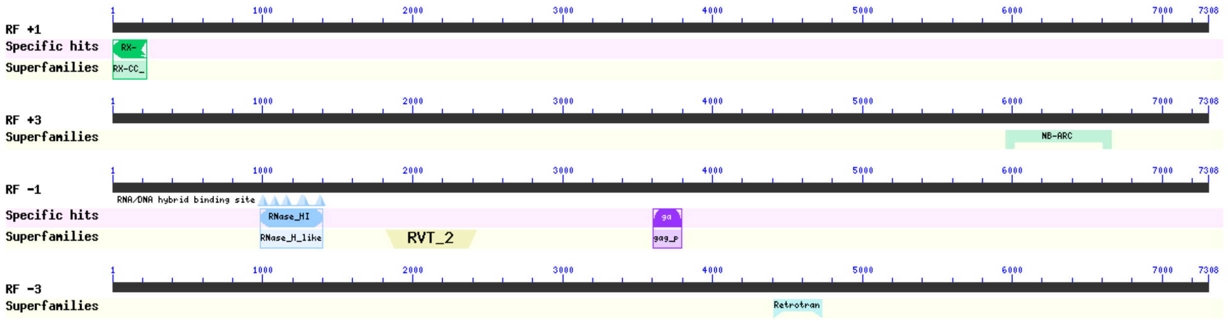

**Chr_10_nlr_19_Ce complete (pseudogene)**				
**Name**	**Accession**	**Description**	**Interval**	**E-value**
RX-CC_like	cd14798	Coiled-coil domain of the potato virus X resistance protein and similar proteins; The potato	1-225	8.90e-19
NB-ARC super family	cl26397	NB-ARC domain	5955-6662	2.74e-43
RNase_HI_RT_Ty1	cd09272	Ty1/Copia family of RNase HI in long-term repeat retroelements; Ribonuclease H	986-1399	1.04e-62
RVT_2 super family	cl06662	Reverse transcriptase (RNA-dependent DNA polymerase)	1823-2422	1.44e-40
gag_pre-integrs	pfam13976	GAG-pre-integrase domain; This domain is found associated with retroviral insertion elements	3602-3796	2.47e-09
Retrotran_gag_2 super family	cl26047	gag-polypeptide of LTR copia-type; This family is found in Plants and fungi	4407-4730	2.75e-10

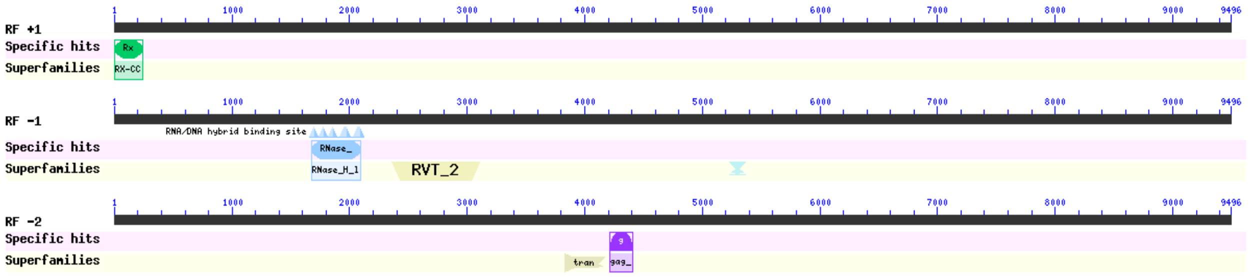

**Chr_1_nlr_53_Ce complete (pseudogene)**				
**Name**	**Accession**	**Description**	**Interval**	**E-value**
Rx_N	pfam18052	This entry represents the N-terminal domain found in many plant resistance proteins	1-243	8.01e-10
RVT_2 super family	cl06662	Reverse transcriptase (RNA-dependent DNA polymerase)	2363-3100	2.71e-68
RNase_HI_RT_Ty1	cd09272	Ty1/Copia family of RNase HI in long-term repeat retroelements; Ribonuclease H	1676-2089	1.39e-64
Retrotran_gag_2 super family	cl26047	gag-polypeptide of LTR copia-type; This family is found in Plants and fungi	5228-5365	4.61e-07
transpos_IS481 super family	cl41329	IS481 family transposase	3826-4164	1.06e-15
gag_pre-integrs	pfam13976	GAG-pre-integrase domain; This domain is found associated with retroviral insertion elements	4216-4407	1.18e-12

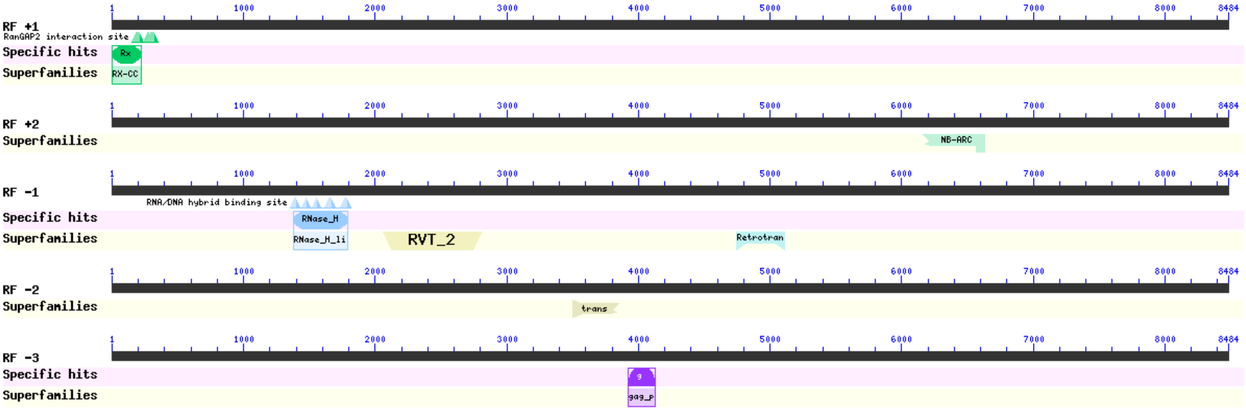

Chr_8_nlr_53 complete (pseudogene)

Name	Accession	Description	Interval	E-value
Rx_N	pfam18052	This entry represents the N-terminal domain found in many plant plant resistance proteins	4-225	1.00e-16
NB-ARC super family	cl26397	NB-ARC domain	6167-6631	6.51e-24
RVT_2 super family	cl06662	Reverse transcriptase (RNA-dependent DNA polymerase)	2062-2802	1.81e-65
RNase_HI_RT_Ty1	cd09272	Ty1/Copia family of RNase HI in long-term repeat retroelements; Ribonuclease H	1381-1791	2.54e-57
Retrotran_gag_2 super family	cl26047	gag-polypeptide of LTR copia-type; This family is found in Plants and fungi	4744-5106	2.87e-22
transpos_IS481 super family	cl41329	IS481 family transposase;	3507-3848	3.91e-10
gag_pre-integrs	pfam13976	GAG-pre-integrase domain; This domain is found associated with retroviral insertion elements	3923-4126	4.06e-13

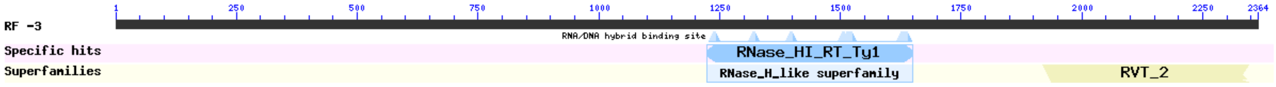

Chr_8_nlr_67_Ce partial (pseudogene)

Name	Accession	Description	Interval	E-value
RNase_HI_RT_Ty1	cd09272	Ty1/Copia family of RNase HI in long-term repeat retroelements; Ribonuclease H (RNase H)	1224-1649	7.59e-41
RVT_2 super family	cl06662	Reverse transcriptase (RNA-dependent DNA polymerase)	1920-2345	8.89e-26

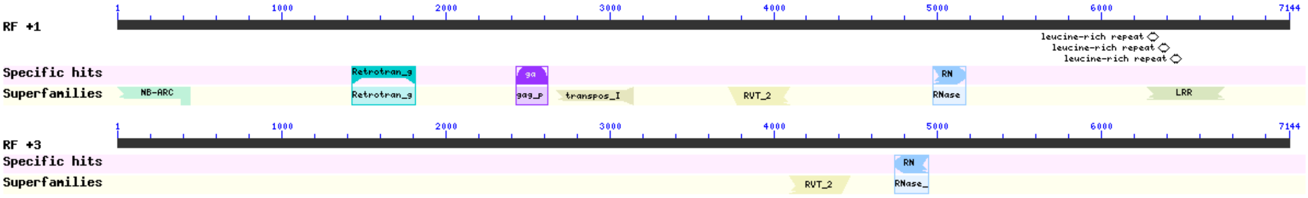

Chr_11_nlr_129_Ce partial (pseudogene)

Name	Accession	Description	Interval	E-value
RVT_2 super family	cl06662	Reverse transcriptase (RNA-dependent DNA polymerase)	3721-4095	1.01e-43
Retrotran_gag_2	pfam14223	gag-polypeptide of LTR copia-type; This family is found in Plants and fungi	1429-1815	2.51e-29
NB-ARC super family	cl26397	NB-ARC domain	1-438	5.97e-26
RNase_HI_RT_Ty1	cd09272	Ty1/Copia family of RNase HI in long-term repeat retroelements; Ribonuclease H	4972-5169	5.72e-23
transpos_IS481 super family	cl41329	IS481 family transposase	2674-3144	4.64e-18
gag_pre-integrs	pfam13976	GAG-pre-integrase domain; This domain is found associated with retroviral insertion elements	2431-2622	5.34e-10
LRR super family	cl34836	Leucine-rich repeat (LRR) protein [Transcription]	6277-6747	2.28e-07
RNase_HI_RT_Ty1	cd09272	Ty1/Copia family of RNase HI in long-term repeat retroelements; Ribonuclease H	4737-4943	4.01e-30
RVT_2 super family	cl06662	Reverse transcriptase (RNA-dependent DNA polymerase)	4095-4466	5.86e-23

Supplementary Figure 2. Conserved domains analysis for loci from NLR-annotator that did not present homology to NLRs proteins by BLASTx analysis. For the analysis, nucleotide sequences were used. The table shows the list of domain hits found for each of the sequences and the domains related to NLR proteins are highlighted in green. The conserved domains were detected using NCBI Conserved Domain Database and the graphical summary was set to concise view. If the alignment omitted more than 20% of the either the n- or c-terminus or both, the partial nature of the hit is indicated in the graphical display as domain with jagged edges. At the end of each ID, the source genome is identified, Ca: *C. arabica*, Cc: *C. eugenioides* and Ce: *C. eugenioides*. RF: reading frame.
